# Clinical and cost-effectiveness of the iStep-MS physical activity and sedentary behaviour intervention for managing fatigue in people with multiple sclerosis: protocol for a multicentre randomised controlled trial

**DOI:** 10.1136/bmjopen-2026-121358

**Published:** 2026-07-20

**Authors:** Daniel P Bailey, Emma Norris, Myles D LeWarne, Luke Cerexhe, Nana Anokye, Amrit Banstola, Joe Gwatsvaira, Meriel Norris, Jennifer Ryan, Beth Stuart, Ann Thomson, Cherry Kilbride

**Affiliations:** 1 Centre for Physical Activity in Health and Disease, College of Health, Medicine and Life Sciences, Brunel University of London, Uxbridge, England, UK; 2 Department of Sport, Health and Exercise Sciences, Brunel University of London, Uxbridge, UK; 3 Department of Health Sciences, College of Health, Medicine and Life Sciences, Brunel University of London, Uxbridge, UK; 4 Centre for Evaluation and Methods, Wolfson Institute of Population Health, Queen Mary University of London, London, England, UK; 5 College of Health and Life Sciences, Brunel University of London, Uxbridge, UK; 6 School of Physiotherapy, Royal College of Surgeons in Ireland, Dublin, Ireland; 7 Wolfson Institute of Population Health, Queen Mary University of London, London, UK

**Keywords:** Multiple sclerosis, Fatigue, Exercise, Randomized Controlled Trial, SPORTS MEDICINE

## Abstract

**Introduction:**

Regular physical activity and limiting sedentary behaviour are important aspects in managing multiple sclerosis (MS). Fatigue is a common and disabling symptom in MS, contributing to impairments in activities of daily living and poorer quality of life. This study aims to determine the effectiveness of a physical activity and sedentary behaviour intervention, called iStep-MS, for reducing fatigue in people with MS when delivered across the MS care pathway.

**Methods and analysis:**

This is a multicentre, two-arm randomised controlled superiority trial with embedded economic and process evaluations. The study will take place across South-East England in acute and community National Health Service settings and charity-funded MS and neurological therapy centres. Intervention deliverers will include a range of healthcare staff such as physiotherapists, occupational therapists, therapy assistants, nurses and exercise therapists. A target sample size of n=198 participants will be randomised 1:1 to the intervention (iStep-MS behaviour change intervention plus usual care) or control (usual care only) arms. Participants will be adults with any type of MS, experiencing MS-related fatigue, relapse-free for >3 months, with a Self-Reported Disability Status Scale category of ≤3.5 (no disability to moderate disability) or 4–6.5 (significant disability). The iStep-MS intervention includes four one-to-one consultation sessions in-person or online with an intervention deliverer over 3 months, incorporating behaviour change techniques aimed at increasing physical activity and reducing sedentary behaviour. The consultations are supported by a handbook designed to help individuals with MS set goals and achieve behavioural changes and a wearable activity tracker for self-monitoring. Outcomes (assessed at baseline, 3 months and 9 months) include self-reported fatigue, quality of life, MS-impact, walking capability, pain, self-efficacy and waist circumference. Sitting, standing and stepping will be measured over 8 days using the activPAL4 device. A process evaluation will assess intervention acceptability, adherence and fidelity, including questionnaires and focus groups with participants and deliverers. A cost-effectiveness analysis will evaluate the value for money of the intervention against usual care.

**Ethics and dissemination:**

Ethical approval has been granted by the NHS London—Bloomsbury Research Ethics Committee (reference 25/LO/0272). Results will be disseminated in scientific journals, conferences and to the wider public (eg, newsletters and social media).

**Trial registration number:**

ISRCTN16944301.

**STRENGTHS AND LIMITATIONS OF THIS STUDY**

- This is the first powered multicentre randomised controlled trial evaluating the effectiveness of a physical activity and sedentary behaviour intervention for reducing fatigue in individuals with multiple sclerosis (MS).
- The intervention is grounded in theory and utilises effective behaviour change techniques for the target behaviours.
- The intervention is delivered by different healthcare staff across the care pathway, optimising its accessibility to individuals with MS.
- An economic evaluation will assess cost-effectiveness of the intervention to inform its uptake into healthcare systems.
- A potential limitation includes the subjective measurement of fatigue and secondary health outcomes.

## Introduction

### Background

 Multiple sclerosis (MS) is a major cause of neurological disability, affecting approximately 1.89 million people worldwide.^1^ Fatigue is the most common and disabling symptom in MS, contributing to impairments in activities of daily living and poorer quality of life (QoL).^2^ Fatigue is estimated to affect 37%–96% of people with multiple sclerosis (PwMS).^3 4^ Physical activity has specific benefits for PwMS including reduced fatigue, increased muscle strength and endurance, milder disability, better walking performance and physical function and improved mental health.^5–8^ In light of these benefits, exercise is recommended in the clinical management of MS.^9^

Despite the benefits, PwMS face challenges with being physically active such as fatigue, impairment and disability, and accessibility.^10^ Therefore, this group engage in less physical activity, accumulating 5840 steps/day on average, which is 3845 steps/day less than the general population.^11 12^ PwMS generally engage in high amounts of sedentary behaviour (mean of 9 hours/day).^13^ Increased sedentary behaviour is adversely associated with fatigue, disability status, walking performance and function in MS.^14–16^ Physical activity and sedentary behaviour are independently associated with MS-related outcomes;^14–16^ therefore, each of these behaviours should be targeted for optimal outcomes in MS, including fatigue management.

There is evidence that interventions comprising self-monitoring of steps and behavioural support may be effective for increasing physical activity in PwMS. For example, self-reported leisure-time physical activity increased over a 6 week pilot intervention that included monitoring steps with a pedometer alongside receiving information and motivation from newsletters and phone calls with research assistants.^17^ A 6 month online intervention using pedometers and web-based sessions with a behavioural coach improved fatigue and self-reported leisure-time physical activity, but there was no change in device-assessed moderate-to-vigorous physical activity.^18^ A systematic review of interventions in PwMS found that the majority of studies measured physical activity via self-report and produced mixed findings regarding effectiveness.^19^ As self-report methods weakly correlate with device-measured physical activity in PwMS and people without MS,^20 21^ device methods are recommended for intervention evaluation.^22^ Yet, interventions have had limited effects on device-assessed physical activity in PwMS. Only one of the six reviewed studies reported an improvement,^19^ which was a 689 steps per day increase at 3 months that was not maintained at 9 months.^5^ A meta-analysis of interventions in PwMS found increased device-assessed physical activity over the short-term (≤12 weeks), but with small effects (standardised mean difference=0.26); and limited long-term (>12 weeks) follow-ups.^23^ To better understand the effectiveness of interventions in PwMS on physical activity, longer term follow-ups with device-assessed outcomes are needed.

The effects of interventions on sedentary behaviour in PwMS are unclear. A systematic review of randomised controlled trials (RCTs) identified only two interventions that evaluated sedentary behaviour outcomes in PwMS.^19^ A 6 month internet-based behaviour change intervention reduced self-reported sitting by 129 min/day,^24^ whereas self-reported sitting was not changed with a 6 month DVD exercise intervention.^25^ These studies are limited by self-report, which may underestimate sedentary behaviour by ≥2.2 hours/day compared with device methods.^26^ Key knowledge gaps remain regarding the effectiveness of interventions targeting sedentary behaviour due to limited literature, self-report sedentary behaviour outcome measures and uncontrolled designs. Furthermore, there is limited evidence concerning the effectiveness of physical activity and sedentary behaviour interventions for reducing fatigue in PwMS.

A randomised controlled feasibility trial exploring a novel intervention targeting physical activity and sedentary behaviour in PwMS addressed previous limitations in the evidence.^27^ The intervention (iStep-MS) was developed to deliver behaviour change techniques (BCTs) promoting self-management. Participants received four face-to-face consultations over 3 months with a physiotherapist or physiotherapy assistant at an MS Therapy Centre, supported by a handbook and a pedometer for self-monitoring of steps.^27^ The study included longer term follow-ups (3 and 9 months) and measured physical activity and sedentary behaviour using device methods. The trial was found to be feasible and the intervention was acceptable for PwMS and intervention deliverers. There were significant increases in stepping by 10.2 min/day at 3 months and 16.2 min/day at 9 months compared with the control group, leading to significant reductions in fatigue of –6.19 (95% CI –12.06 to –0.31) and –8.97 (95% CI –16.21 to –1.72), respectively.^27^ QoL, MS self-efficacy and autonomy were also significantly improved. Based on these findings, the clinical effectiveness of iStep-MS for improving fatigue should be evaluated in a definitive RCT.

Despite evidence of physical activity and sedentary behaviour interventions being effective in PwMS, including iStep-MS, key limitations are delivery within a single setting (eg, in a specific MS service or online) or by a single profession (eg, behavioural coaches, physiotherapists or non-specialist interventionists),^19 23 27 28^ limiting generalisability and accessibility to PwMS. Understanding the effectiveness of iStep-MS when delivered by different healthcare staff across different services in the MS care pathway would significantly advance knowledge and provide evidence for a scalable and accessible intervention. Furthermore, the cost-effectiveness of physical activity and sedentary behaviour interventions in PwMS has not been determined. Demonstrating value for money will better inform healthcare and stakeholder resource allocation decisions to support wider provision and reach of such interventions.

The primary aim of this study is to determine the effectiveness of a physical activity and sedentary behaviour intervention, iStep-MS, for reducing fatigue in PwMS when delivered across the MS care pathway. Secondary aims are to determine cost-effectiveness of iStep-MS and investigate effects on sitting, standing and stepping, walking capability, pain, impact of MS, self-efficacy and QoL.

## Methods and analysis

### Study design

**Figure 1:**
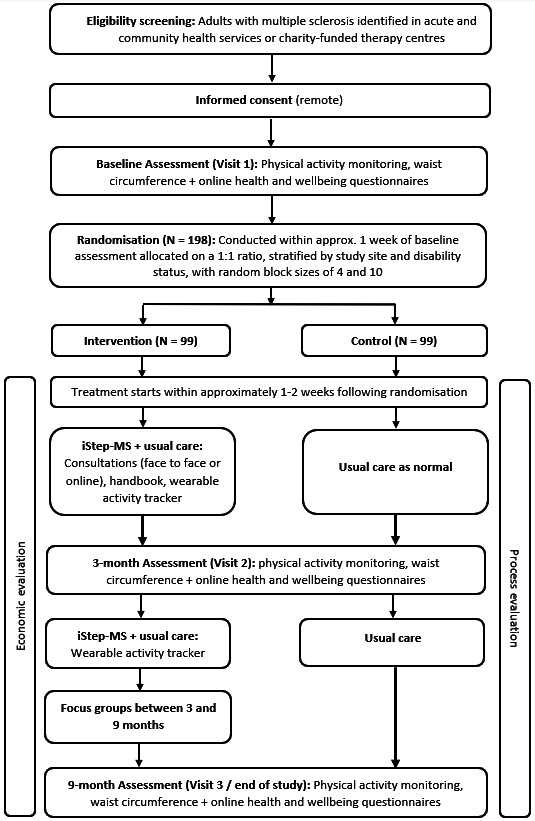
Study flow diagram. MS, multiple sclerosis.

This is an unblinded, two-arm, multicentre randomised controlled superiority trial with a target sample of 198 participants (99 per arm). Following baseline measures, participants will be individually randomised to (1) iStep-MS plus usual care (intervention group), or (2) usual care only (control group). Outcome measurements will be repeated at 3 and 9 months after randomisation. The study protocol is reported following the Standard Protocol Items: Recommendations for Interventional Trials (SPIRIT) checklist.^29^ See online supplemental material 1 for schedule of enrolment, interventions and measurements. The study flow diagram is shown in figure 1.

### Study setting

The study will take place across the MS care pathway in South-East England including acute and community National Health Service (NHS) settings, and charity-funded MS and neurological therapy centres. The sites will be located within city and suburban areas in regions with diverse ethnic demography.

### Study population

Individuals eligible for this study include adults aged ≥18 years with any type of MS. Participants will have experienced MS-related fatigue over the past 4 weeks, as indicated by a score of >35.5 for total Modified Fatigue Impact Scale (MFIS) score, >18.5 for combined physical and social subscales and/or >15.5 for cognitive subscale.^30^ Other inclusion criteria include being relapse-free for 3 months prior to consenting into the study, no contraindications to participating in physical activity and a score of ≤3.5 (no disability to moderate disability) or 4.0–6.5 (significant disability) on the Self-Reported Disability Status Scale (SRDSS).^31^ The SRDSS is validated for assessing mobility aspects of the Expanded Disability Status Scale (EDSS), with the SRDSS categories being used in this study aligning to EDSS scores of 1.0 ‘no disability, very small sign that one function isn’t normal’ to 6.5 ‘you can walk 20 metres with two aids (e.g. crutches, sticks, frame, etc) without stopping for rests’.^31^ Additionally, participants must be able to communicate in English and travel to a study site or attend online consultations for the intervention. Exclusion criteria are an SRDSS category of ≥7.0,^31^ pregnancy, currently participating in a clinical trial, or lacking capacity to provide informed consent.

### Sample size

Sample size was calculated based on the primary outcome, fatigue. A minimum clinically important difference of 8.11 on the MFIS^32^ and a SD of 18.1 from the iStep-MS feasibility study^27^ results in an effect size of d=0.45. To detect this effect with 80% power and α=0.05, n=158 would be required. To account for 20% dropout, a target of 198 participants (n=99 in each arm) will be recruited.

### Participant recruitment

Participants will be recruited from the study sites from August 2025 to April 2026. The study information will be advertised at sites through newsletters, posters and leaflets, dedicated study webpages on the sponsor (Brunel University of London) and the MS Society (a national charity and funder of this research) websites, and mail-out to volunteers who have registered with the Be Part of Research Volunteer Service (delivered by the National Institute for Health and Care Research) and meet the study eligibility criteria. The research team will also undertake in-person recruitment at non-NHS study sites, where appropriate, and promote the study through local MS support groups. Potential participants will have initial eligibility checked (diagnosis of MS, pregnancy, contraindications to physical activity, able to walk 10 m and participation in a clinical trial) by the relevant study site and will then be put in touch with the central research team for full eligibility assessment. Individuals will also be able to express their interest in taking part by completing a short online survey or contacting the research team by email or phone, followed by eligibility assessment. On confirmation of eligibility, participants will provide written or electronic informed consent.

### Randomisation

Participants will be randomised following baseline measures on a 1:1 ratio to the intervention arm (iStep-MS plus usual care) or control arm (treatment as usual). Allocation will be performed by a study researcher using a validated web-based system (REDCap). Random permuted blocks of sizes 4 and 10 will be used, with stratification by study site and SRDSS category grouped as ≤3.5 (no disability to moderate disability) and 4–6.5 (significant disability).^31^

### iStep-MS intervention

Each participant will receive the iStep-MS intervention for 3 months alongside usual care. As the feasibility study found that iStep-MS was safe and acceptable,^27^ the intervention will be delivered using similar procedures as before, but with improvements informed by feedback from previous participants and Patient and Public Involvement (PPI). Intervention participants will be sent an iStep-MS handbook and a wearable activity tracker (Fitbit Charge 6) by post, which will support the intervention consultation sessions and maintenance of behaviour change thereafter. Table 1 specifies the BCTs (using the BCT Taxonomy v1 (BCTTv1)^33^), modes of delivery (using the Modes of Delivery Ontology^34^) and source (who delivers, using the Intervention Source Ontology^35^) implemented across the iStep-MS intervention.

**Table 1:** Behaviour change techniques, mode of delivery and source within the iStep-MS intervention

Programme component	Behaviour change techniques	Mode of delivery	Source
Consultations (guided by Handbook)	Feedback on behaviour (BCT 2.2) Social support (unspecified) (BCT 3.1)	Individual-based (BCIO:011055) Face to face (BCIO:011003) At-a-distance (BCIO:011004) Audio call (BCIO:011022) Video call (BCIO:011023)	Physiotherapist (BCIO:010021) Physiotherapy technician and assistant (BCIO:010053) Health associate professional (BCIO:010045)
Handbook	Goal setting (behaviour) (BCT 1.1) Problem solving (BCT 1.2) Action planning (BCT 1.4) Review behaviour goal(s) (BCT 1.7) Self-monitoring of behaviour (BCT 2.3) Instruction on how to perform the behaviour (BCT 4.1) Information about health consequences (BCT 5.1) Graded tasks (BCT 8.7) Focus on past success (BCT 15.3)	Printed publication (BCIO:011008)	Physiotherapist (BCIO:010021) Physiotherapy technician and assistant (BCIO:010053) Health associate professional (BCIO:010045)
Wearable Activity Tracker	Self-monitoring of behaviour (BCT 2.3) Biofeedback (BCT 2.6) Adding objects to the environment (BCT 12.5)	Wearable electronic device (BCIO:011015)	Physiotherapist (BCIO:010021) Physiotherapy technician and assistant (BCIO:010053) Health associate professional (BCIO:010045)

Behaviour change techniques are characterised using the Behaviour Change Techniques Taxonomy v1 (BCTTv1). Modes of delivery are characterised using the Mode of Delivery Ontology. Intervention Source is characterised using the Intervention Source Ontology.

BCT, behaviour change technique; MS, multiple sclerosis.

### iStep-MS consultations

There will be four one-to-one physical activity and sedentary behaviour consultation sessions delivered to each participant approximately 1, 3, 7 and 11 weeks after randomisation. The sessions are expected to last approximately 45–60 min. Indicative content of the sessions is shown in box 1. Intervention deliverers will be healthcare staff with varying qualification levels who provide MS care at the study sites. Job roles may include physiotherapists, occupational therapists, therapy assistants, nurses and exercise professionals. All deliverers will receive behaviour change training from the research team specific to the iStep-MS intervention. It is intended that participants will be supported by the same deliverer throughout all consultations where possible, to create a supportive environment, build rapport and improve confidence.^27^ Consultations will take place in-person or via online video call depending on the options each site is able to offer and participant preference.

Box 1 **Indicative content for the iStep-MS consultation sessions**

**Consultation 1 (intervention start): first steps**

Consultation 1 will occur within approximately 10 days after randomisation. The handbook will contain sedentary behaviour, physical activity and steps diaries. The deliverer will discuss the participant’s physical activity and sedentary behaviour, including consideration of data provided by the wearable activity tracker (BCT 2.3: Self-monitoring of behaviour; BCT 2.6: Biofeedback; BCT 12.5: Adding objects to the environment). This information will be used as a starting point to inform the physical activity journey planner. Further techniques used by the deliverer during this consultation will include education regarding current sedentary behaviour and physical activity guidelines and levels of activity intensity relevant to MS (BCT 4.1: Instruction on how to perform the behaviour), identification of potential benefits and importance of limiting sedentary behaviour and increasing physical activity (BCT 5.1: Information about health consequences), planning and setting sedentary behaviour, physical activity and step-count goals (BCT 1.1: Goal setting (behaviour)).

**Consultation 2: overcoming challenges**

Consultation 2 will occur approximately 3 weeks after randomisation. Progress and individualised goals from consultation 1 will be reviewed using the sedentary behaviour, physical activity and steps diaries (BCT 1.7: Review behaviour goals; BCT 2.2: Feedback on behaviour). Barriers and facilitators to progress will be discussed (BCT 1.2: Problem solving). Strategies to overcome barriers will be identified and positive coping strategies will be developed (BCT 1.2: Problem solving; BCT 3.1: Social support (unspecified)). Goals will be reviewed and progressed as appropriate (BCT 1.1: Goal setting (behaviour); BCT 1.7: Review behaviour goals).

**Consultation 3: keeping up the changes**

Consultation 3 will occur approximately 7 weeks after randomisation. Progress and individualised goals from consultation 2 will be reviewed using the sedentary behaviour, physical activity and steps diaries (BCT 1.7: Review behaviour goals; BCT 2.2: Feedback on behaviour). Setbacks will be discussed and strategies for overcoming them will be developed (BCT 1.2: Problem solving). Education about pacing and prioritisation of activity will be provided (BCT 4.1: Instruction on how to perform the behaviour). If-then plans will be established to support habit building (BCT 1.4: Action planning). Goals will be reviewed and progressed as appropriate (BCT 1.1: Goal setting (behaviour); BCT 1.7: Review behaviour goals).

**Consultation 4: building lasting habits**

Consultation 4 will occur approximately 11 weeks after randomisation. Progress and individual goals from consultation 3 will be reviewed using the sedentary behaviour, physical activity and steps diaries (BCT 1.7: Review behaviour goals; BCT 2.2: Feedback on behaviour). Overall progress and personal benefits of participation in the intervention will be reviewed (BCT 15.3: Focus on past success). Strategies for increasing and maintaining changes in sedentary behaviour and physical activity over the long term will be discussed (BCT 1.4: Action planning; BCT 8.7: Graded tasks), in addition to developing habits in the case of relapsing or progressively worsening illness (BCT 1.2: Problem solving). Self-monitoring skills will be developed (BCT 2.3: Self-monitoring of behaviour). New long-term goals will be set (BCT 1.1: Goal setting (behaviour)).

BCT, behaviour change technique.

### iStep-MS handbook

Cognitive-behavioural trainers with experience in training healthcare professionals to use brief BCTs, the research team and an advisory group of PwMS and neurological physiotherapists developed the iStep-MS handbook.^27^ The development process involved adapting the NHS Health Trainer Handbook, which incorporated established techniques to support changing behaviours that optimise self-management and prevent ill-health. Within the handbook, there is content dedicated to each of the four consultation sessions, which includes (1) session overview, (2) presession reading and reflection, (3) session-specific content, for example, barriers and facilitators for physical activity, (4) goal setting and (5) a diary to record and monitor goals.

### Wearable activity tracker

A wrist-worn Fitbit Charge 6 will be provided to each participant to support self-monitoring of physical activity (steps and active minutes) and sedentary behaviour (prompts to move following periods of inactivity) during the intervention. In the iStep-MS feasibility study, participants were provided with a hip-worn pedometer to monitor and record their step-count. Although this approach to self-monitoring steps and supporting progress with goals motivated participants to initiate positive health changes,^36 37^ there were usability issues with the pedometer. These included being unable to attach it to clothing (reducing confidence in accuracy) and detachment during strenuous physical activity and other activities like toileting. We conducted a follow-up study in PwMS to explore acceptability and experiences of different wearable activity trackers. A wrist-worn device that had good visibility (well-sized, well-lit and easy-to-read screens), was perceived as accurate, enabled goal setting, provided prompts to move and could be easily attached for charging were important considerations.^38^ Informed by these findings and PPI feedback, the Fitbit Charge 6 was selected for this study.

There is conflicting evidence regarding validity of Fitbit devices for measuring physical activity during activities of daily living,^38^ but this device had good agreement with criterion measures for steps during walking tasks and moderate-excellent agreement for time in moderate-to-vigorous physical activity and total daily physical activity in PwMS.^39^ The research team will provide support with device setup by phone or video call, if required, prior to their first iStep-MS consultation session. Informed by PPI feedback, participants can instead use their own wearable activity tracker (as this likely reflects acceptance and trust in the device) if it provides information on steps and sends inactivity alerts to prompt movement.

### Intervention deliverer training

Intervention deliverers will receive two half days of training from the research team prior to intervention delivery, with a ‘booster’ session during the intervention. The training will include guidance on using the iStep-MS handbook, introduce the COM-B (Capability, Opportunity, Motivation—Behaviour) model of behaviour change,^40^ and cover principles of Cognitive Behavioural Therapy and Motivational Interviewing. Experiential activities will be used to develop and refine intervention delivery, including timing and pacing of sessions. A training handbook will be provided to all intervention deliverers including the training activities and note-taking areas.

### Control group

Control group participants will continue with usual care as normal. Usual care provided may range from no treatment to multidisciplinary holistic care (eg, exercise prescription, massage and oxygen therapy) and, for acute care sites, may also include disease-modifying treatments. Both intervention and control group participants will be asked not to discuss the study with other participants or patients to minimise contamination. To further reduce contamination and encourage engagement with the study, control participants will be provided with the intervention handbook and a Fitbit Charge 6 at the end of the study.

### Study measurements

Data collection will take place remotely to increase accessibility to the study and reduce participant burden. Study measurements will occur at baseline, 3 months post randomisation and 9 months post randomisation. ‘Assessment packs’ will be sent to participants for undertaking these measures consisting of an activPAL4 activity monitor (PAL Technologies, Glasgow, UK) with instructions and an accompanying activity diary, a tape measure with instructions for measuring waist circumference and a pre-paid envelope for returning equipment. Participants will be provided with a £20 shopping gift voucher after completing study measurements at each timepoint.

#### Primary outcome (fatigue)

The primary outcome, fatigue, will be measured using the MFIS, which assesses physical, psychological and cognitive effects of fatigue.^41 42^ The MFIS has high internal consistency, excellent test–retest reliability and good construct validity in PwMS.^27 42^ The MFIS items will be aggregated into physical, cognitive and psychosocial subscale scores (with ranges of 0–36, 0–40 and 0–8, respectively), and a total MFIS score (range of 0–84). Higher scores indicate greater impact of fatigue.

#### Secondary outcomes

##### Sitting, standing and stepping

An activPAL4 activity monitor will be worn on the anterior aspect of the thigh at each timepoint to measure daily sitting, standing and stepping time, prolonged sitting, number of steps and number of sit-to-upright transitions over seven full consecutive days. The activPAL provides valid data for these outcomes in PwMS.^43 44^ Sleep and wake times will be recorded by participants in a diary, in addition to times that the activPAL is removed. ActivPAL data will be downloaded using proprietary software (PAL Technologies, Glasgow, Scotland) and processed with Processing PAL V1.41—21022022 (University of Leicester, UK) to identify valid waking wear data.^45^ Criteria to confirm a valid wear day will be ≥10 hours of waking wear time, <95% of time spent in a single event (ie, sitting, standing or stepping) and ≥250 steps.

##### Waist circumference

An adjustable tape measure will be used by participants to measure their waist circumference three times to the nearest 0.1 cm at the level of their umbilicus following gentle expiration. The mean of these three measures will be used for analysis.

##### Quality of life

The EuroQol 5 Dimension 5 Level (EQ-5D-5L) will be used to assess QoL across five dimensions: mobility, self-care, usual activities, pain/discomfort and anxiety/depression.^46 47^ The EQ-5D-5L provides good discriminatory capacity in MS.^46^ Responses will be answered using a 5-point Likert scale, which will be used to derive index values for each dimension and an overall England-specific value set. Overall health will be rated on a 0–100 visual analogue scale.^47^ Higher scores reflect greater QoL across all items.

##### Impact of MS

The physical and psychological impact of MS during the past 2 weeks will be assessed using the 29-item Multiple Sclerosis Impact Scale (MSIS-29).^48^ The MSIS-29 has good to excellent reliability and demonstrated construct validity with other measures of physical and psychological health in PwMS.^48^ Physical and psychological impact scores are calculated by summing their respective items and then transforming to a score on a scale of 0–100. Higher scores on the MSIS indicate greater impact of MS on day-to-day life.

##### Walking capability

The Twelve Item MS Walking Scale (MSWS-12) will be used to measure balance, use of support, speed, distance and automaticity aspects of walking capability over the past 2 weeks. This questionnaire has excellent validity and test–retest reliability, and high internal consistency across a range of disability severities in PwMS.^49^ The item responses are summed, giving a total out of 60 and then transformed on a scale of 0–100. A higher MSWS-12 score reflects poorer walking capability.

##### Pain

Pain will be assessed using the pain section of the EQ-5D-5L on a 5-point Likert scale, with higher scores indicating more pain.

##### Self-efficacy

The Multiple Sclerosis Self-Efficacy Scale (MSSE) measures confidence in behaviours for Activities of Daily Living and managing disease symptoms, reactions to MS-related limitations, and impact of MS on life activities.^50^ The MSSE has good construct validity and test–retest reliability.^50^ Item scores are summed to create a self-efficacy score for Function, Symptom and Control subscales. A total score is also created with higher scores indicating greater self-efficacy.

### Statistical analysis

Baseline characteristics for each group and the whole sample will be described descriptively. The primary analysis will be ‘intention-to-treat’, analysing participants with available outcome data in their randomised groups regardless of adherence to the trial. The primary analysis will identify if the intervention and control lead to a difference in MFIS score at 9 months post randomisation. Differences in MFIS scores between groups will be estimated using a linear regression model, adjusting for stratification factors, baseline MFIS score and other relevant prognostic factors. Potential clustering with randomising sites will be accounted for using robust SEs. Adjusted mean differences between groups will be presented with 95% CI and p values. The pattern of missingness will be examined and, if appropriate, a sensitivity analysis will be undertaken using a multiple imputation model for missing data.

Secondary outcomes at 3 and 9 months will be analysed using regression models appropriate to the outcome distribution (eg, linear for continuous data, logistic for binary data), controlling for the baseline level of the outcome (where collected), stratification factors and key prognostic factors, as per the primary outcome. The effect of non-adherence (<70% completion of consultations) with the allocated intervention will be evaluated using per-protocol analyses.

### Health economic evaluation

The economic evaluation will involve cost-effectiveness analysis (from baseline to 9 months) to examine value for money of iStep-MS against usual care. The perspective for the analysis will be NHS and personal social services, and patient.^51^ The resource use will comprise: (a) training for deliverers; (b) consultations provided by deliverers; (c) handbooks and wearable trackers and (d) health and personal social service use via a modified Client Service Receipt Inventory (CSRI),^52^ which will be completed by participants at baseline, 3 and 9 months. Completion of the CSRI has been validated against General Practitioner case records.^53^ Out of pocket expenses related to physical activity sessions (eg, transport costs) will be collected via self-report at baseline, 3 and 9 months. Resource use data will be collected through interviews with trial and intervention staff (including collecting information on staff time and money spent on each activity) and review of trial management records. Unit costs will be taken from the NHS reference costs,^54^ standard unit costs^55^ and published literature.

The key outcome will be an incremental cost per Quality-Adjusted Life-Year (QALY), which will be based on the EQ-5D-5L. There will also be exploratory analysis of QALY estimation based on MS-specific health states using the eight-dimension Multiple Sclerosis Impact Scale (MSIS-8D), which is derived from the MSIS-29.^56^ Cost per change in fatigue will also be measured. Analyses will show mean total costs and mean utilities by trial arm and differences between trial arms. Uncertainty will be represented in cost-effectiveness acceptability curves for intervention vs control. Subgroup analysis will be undertaken using the trial stratification factors. If other factors are found to be significant in the subgroup statistical analyses, then it will be considered if it is possible to use these and whether there is a good economic/ policy reason to do so.

### Process evaluation

A mixed-methods process evaluation will be undertaken to explore intervention fidelity, adherence and acceptability. Fidelity of intervention implementation will be evaluated following the fidelity in complex rehabilitation interventions guidelines.^57 58^ Participant attendance at iStep-MS consultation sessions will be recorded by intervention deliverers. At least 10% of each intervention deliverer’s consultations will be audio-recorded and rated by a researcher against a checklist for content delivery (online supplemental material 2). Delivery of ≥70% of expected content will be considered acceptable.^58^ Fidelity to delivery skills will be assessed and considered acceptable if competence is scored at level 2 (‘Evidence of competence, but lack of consistency’) to 6 (‘Excellent performance, effective management of difficulties’) in each skill category. The percentage of handbook sections completed by participants will be assessed against a checklist (online supplemental material 3) using photographs from all handbook sections for ≥20% of intervention participants.

A subsample of intervention participants (n=24) and deliverers (n=12) from across the study sites will take part in focus groups following 3 month measurement and intervention delivery periods to explore acceptability and experiences of taking part in iStep-MS. Focus group topics will be guided by the previous iStep-MS fidelity evaluation^58^ and the Theoretical Framework of Acceptability;^59 60^ see online supplemental material 4. The qualitative data will be analysed using the Framework Method of Analysis.^59^

### Data management

All study documentation containing identifiable participant data will be managed in accordance with ICH–GCP (International Council for Harmonisation–Good Clinical Practice), the UK Policy Framework for Health and Social Care Research, the Data Protection Act 2018 and most recent UK General Data Protection Regulation. Each participant will be allocated a unique study ID that will be used in data records to preserve anonymity. Study paperwork will be stored in locked filing cabinets at the study sites and will only be accessible to authorised study personnel. Study data will be captured electronically in a secure password-protected bespoke REDcap database system. Pseudonymised data for the health economic evaluation will be securely transferred to the health economics researchers for analysis, which will be held in password protected files.

Audio recordings from focus groups and consultation sessions will be made using the recording feature of Microsoft Teams, on a smartphone or tablet or with an encrypted Dictaphone. The recordings will be transcribed using the automatic transcription function of Microsoft Teams or proprietary Dictaphone software and manually checked by a researcher. Transcripts will be anonymised or assigned a pseudonym and saved onto a secure university network server. The audio recordings will then be deleted.

### Trial monitoring

A Trial Steering Committee (TSC) will ensure that appropriate ethical approvals are obtained and will monitor progress of the trial, protocol adherence, substantial protocol amendments and patient safety. The TSC will also receive and review the progress and accruing data of the trial. The Committee will comprise an independent chair, an independent expert in the study area, an independent statistician, a public member with MS and study investigators.

### Adverse event reporting and harms

Any adverse events (AEs) reported by participants, such as falls, illness or skin irritation from the activPAL or Fitbit, will be recorded within the trial database during the study. At the 3 month and 9 month post randomisation timepoints, participants will complete a questionnaire asking if they have experienced a fall, relapse or AE since their last point of contact in the study. If a participant reports an AE directly to their study site, a delegated staff member will report it to the Principal Investigator and central research team for it to be recorded within the trial database. Data recorded on AEs will include date of onset, resolution, outcome, severity and causality. Expectedness and cause (probable, possible, unlikely or unrelated to the trial) of the AE will be assessed by the Principal Investigator or the central research team.

Serious adverse events (SAEs) (AEs which are life-threatening, resulting in hospitalisation, death or prolonged and worsened impairment) should be reported to the Chief Investigator by the site Principal Investigator or researcher within 24 hours of becoming aware of the event. The Chief Investigator will be responsible for notifying the trial Sponsor of an SAE within 48 hours and the NHS Research Ethics Committee that gave a favourable opinion of the research within 15 days of becoming aware of the event. A ‘directly attributable’ SAE will only be recorded and reported if the event occurs during contact time with an intervention deliverer or while undertaking physical activity as part of the intervention. Participants who experience an SAE that affects their continued safety or significantly affects the conduct or outcomes of the study will be withdrawn.

### Patient and public involvement

Patient and public members contributed to the development of this research through advising on aspects related to study design, participant recruitment, data collection and intervention delivery. A Public Advisory Group comprising PwMS and healthcare professionals will advise the research team during the trial on matters relating to direction of the research, participant-facing documents and the public dissemination strategy.

## Ethics and dissemination

This study received ethical approval from the College of Health, Medicine and Life Sciences, Brunel University of London (reference 50434-NHS-Feb/2025-53 798-2) and the NHS London—Bloomsbury Research Ethics Committee (reference 25/LO/0272).

Dissemination to academic audiences will include scientific journal publications and presentations at conferences. The findings will be disseminated to the public and relevant stakeholders via newsletters, a dedicated University Webpage and the MS Society website. To maximise reach and impact of the study findings, dissemination materials will be made open access.

### Data access statement

Datasets generated and/or analysed during the current study will be stored in a publicly available repository: https://brunel.figshare.com/. Quantitative data will be made available for a minimum of 10 years following data analysis. To protect participant confidentiality, supporting focus group transcripts will not be made publicly available.

## Supplementary material

10.1136/bmjopen-2026-121358 online supplemental file 1

10.1136/bmjopen-2026-121358 online supplemental file 2

10.1136/bmjopen-2026-121358 online supplemental file 3

10.1136/bmjopen-2026-121358 online supplemental file 4
