# Supplementary material for: Clinical and cost-effectiveness of the iStep-MS physical activity and sedentary behaviour intervention for managing fatigue in people with multiple sclerosis: protocol for a multicentre randomised controlled trial
Source: BMJ Open. 2026 Jul 20;16(7):e121358. doi: 10.1136/bmjopen-2026-121358 (PMC13386072; doi:10.1136/bmjopen-2026-121358)
Supplement: online supplemental file 1 [file bmjopen-16-7-s001.docx]

**Supplementary Material 1**. iStep-MS study schedule of enrolment, interventions, and assessments

|  | | **Screening** | **Consent** | **Baseline**  **(Visit 1)** | **Randomisation** | **Intervention** | | | | **3 Months**  **(Visit 2)** | **4 months** | **9 Months**  **(Visit 3)** |
| --- | --- | --- | --- | --- | --- | --- | --- | --- | --- | --- | --- | --- |
|  | | | | | | wk1 | wk3 | wk7 | wk11 |  |  |  |
| **Screening and consent** | |  |  |  |  |  |  |  |  |  |  |  |
| Screening | | **X** |  |  |  |  |  |  |  |  |  |  |
| Participant Contact Information | |  | **X** |  |  |  |  |  |  |  |  |  |
| Informed consent | |  | **X** |  |  |  |  |  |  |  |  |  |
|  |  |  |  |  |  |  |  |  |  |  |  |  |
| Demographics and background | |  |  | **X** |  |  |  |  |  |  |  |  |
| MFIS | |  |  | **X** |  |  |  |  |  | **X** |  | **X** |
| activPAL4 | |  |  | **X** |  |  |  |  |  | **X** |  | **X** |
| Waist circumference | |  |  | **X** |  |  |  |  |  | **X** |  | **X** |
| EQ-5D-5L | |  |  | **X** |  |  |  |  |  | **X** |  | **X** |
| MSIS-29 | |  |  | **X** |  |  |  |  |  | **X** |  | **X** |
| MSWS-12 | |  |  | **X** |  |  |  |  |  | **X** |  | **X** |
| MSSE | |  |  | **X** |  |  |  |  |  | **X** |  | **X** |
|  |  |  |  |  |  |  |  |  |  |  |  |  |
| Randomisation | |  |  |  | **X** |  |  |  |  |  |  |  |
|  |  |  |  |  |  |  |  |  |  |  |  |  |
| Intervention resource use | |  |  | **X** |  | **X** | **X** | **X** | **X** |  |  |  |
| Client Service Receipt Inventory | |  |  | **X** |  |  |  |  |  | **X** |  | **X** |
| Cost of physical activity participation | |  |  | **X** |  |  |  |  |  | **X** |  | **X** |
|  |  |  |  |  |  |  |  |  |  |  |  |  |
| Consultation attendance & delivery checklist | |  |  |  |  | **X** | **X** | **X** | **X** |  |  |  |
| Intervention fidelity assessment | |  |  |  |  | **X** | **X** | **X** | **X** |  |  |  |
| Fidelity to handbook checklist | |  |  |  |  |  |  |  |  | **X** |  |  |
| Focus groups | |  |  |  |  |  |  |  |  |  | **X** |  |
|  |  |  |  |  |  |  |  |  |  |  |  |  |
| Events reporting | |  |  |  |  |  |  |  |  | **X** |  | **X** |
