# Supplementary material for: Clinical and cost-effectiveness of the iStep-MS physical activity and sedentary behaviour intervention for managing fatigue in people with multiple sclerosis: protocol for a multicentre randomised controlled trial
Source: BMJ Open. 2026 Jul 20;16(7):e121358. doi: 10.1136/bmjopen-2026-121358 (PMC13386072; doi:10.1136/bmjopen-2026-121358)
Supplement: online supplemental file 2 [file bmjopen-16-7-s002.pdf]

INTERVENTION FIDELITY TOOL		
**PARTICIPANT ID**	**M S -** ☐☐ - ☐☐☐	iStep-MS

Reviewer completes this checklist against audio recording of session.

CONSULTATION ONE: FIRST STEPS	
**Deliverer ID:**	**M S D E -** ☐☐ - ☐☐
**Reviewer (initials):**	
**Date of review:**	D D M M Y Y Y Y

CONTENT OF SESSION	COMPLETED			
	Yes	Partial	No	N/A
1. Review pre-reading and check understanding of activity levels and the process for the intervention.				
2. Find out how MS affects participant personally.				
3. About you – values.				
4. Discuss the benefits and importance of reducing sedentary behaviour and increasing physical activity in people with MS.				
5. Personal benefits of reducing sedentary behaviour and increasing physical activity.				
6. Review of definitions of physical activity and general advice/recommendations. Check understanding.				
7. Review of definitions of sedentary behaviour and general advice/recommendations. Check understanding.				
8. Review of step count as a physical activity and general advice/recommendations. Check understanding.				
9. Discuss current sedentary behaviour and physical activity using information from smartwatch.				
10. Set 3-month targets.				
11. Generate steps to reach targets and a specific goal for next session.				
12. Measure confidence level on the confidence ruler (scale).				
13. Detail planning – facilitate what, where, when, who, how long? Facilitate problem solving.				
14. Recording activity in diary – how, when and additional sheets.				
15. Check participant can use smartwatch to track physical activity and sedentary behaviour (hourly activity monitoring and inactivity alerts).				
16. Teach how to work out average daily step count.				
17. Final check: summarise what has been agreed and check participant understanding.				
18. Plan date/time next session. Remind participant of tasks and to bring workbook.				

INTERVENTION FIDELITY TOOL	
PARTICIPANT ID	M S - [ ][ ] - [ ][ ][ ]

SKILLS	SCORE (0-6)	COMMENTS
**1. Framing, pacing, focus & use of time**<br>• Did the structure and flow of the session facilitate successful use of time?<br>• Were unproductive digressions steered past sensitively?		
**2. Empowering explanations**<br>• Did explanations invite discussion/participation?		
**3. Collaboration and active listening: therapeutic alliance**<br>• Were open questions used?<br>• Was the participant encouraged to actively participate?<br>• Were goals participant led?		
**4. Goal setting and actions**<br>• Were goals set at 3 levels (PA, step and sedentary)?<br>• Did the action planning facilitate problem solving?		
**5. Feedback, reviewing and summarising**<br>• Was praise used appropriately to support change?<br>• Did feedback reinforce change talk?<br>• Was understanding/meaning checked?		
**6. Building self-efficacy**<br>• Was optional use of forms highlighted?<br>• Were opportunities to affirm change talk and action taken?		
**Additional comments:**		

Competence level

Incompetent
Novice
Advanced beginner
Competent
Proficient
Expert

Examples

0	Absence of feature, or highly inappropriate performance
1	Inappropriate performance, with major problems evident
2	Evidence of competence, but lack of consistency
3	Competent, but some inconsistencies
4	Good features, but minor inconsistencies
5	Very good features, very minimal inconsistencies
6	Excellent performance, effective management of difficulties

INTERVENTION FIDELITY TOOL	
PARTICIPANT ID	M S - __ __ - __ __ __

CONSULTATION TWO: OVERCOMING CHALLENGES

**Deliverer ID:**	M S D E - __ __ - __ __
**Reviewer (initials):**	
**Date of review:**	D D M M Y Y Y Y

CONTENT OF SESSION	COMPLETED			
	Yes	Partial	No	N/A
1. Review pre-reading and check understanding of adjustment/flexibility, the role of thoughts and feelings and factors which help to cope with MS.				
2. Opportunity for questions.				
3. Review goals, diary recordings and discuss what went well and what got in the way.				
4. Facilitate discussion on barriers and facilitators for reducing sedentary behaviour and increasing physical activity.				
5. Facilitate discussion on developing positive coping strategies.				
6. Review overall sedentary behaviour and physical activity targets and set new specific goals and record in diary.				
7. Check confidence levels; Build confidence to make change (optional).				
8. Detail planning – facilitate what, where, when, who, how long? Facilitate problem solving.				
9. Discuss if participant wishes to set new goals between sessions (optional).				
10. Final check: summarise what has been agreed and check participant understanding.				
11. Plan date/time next session. Remind participant of tasks and to bring workbook.				

INTERVENTION FIDELITY TOOL

PARTICIPANT ID	M S - ☐☐ - ☐☐☐

SKILLS	SCORE (0-6)	COMMENTS
**1. Framing, pacing, focus & use of time**<br>• Did the structure and flow of the session facilitate successful use of time?<br>• Were unproductive digressions steered past sensitively?		
**2. Empowering explanations**<br>• Did explanations invite discussion/participation?		
**3. Collaboration and active listening: therapeutic alliance**<br>• Were open questions used?<br>• Was the participant encouraged to actively participate?<br>• Were goals participant led?		
**4. Goal setting and actions**<br>• Were goals set at 3 levels (PA, step and sedentary)?<br>• Did the action planning facilitate problem solving?		
**5. Feedback, reviewing and summarising**<br>• Was praise used appropriately to support change?<br>• Did feedback reinforce change talk?<br>• Was understanding/meaning checked?		
**6. Building self-efficacy**<br>• Were they encouraged to come up with own examples?<br>• Were opportunities to affirm change talk and action taken?<br>• Was reflection from pre-reading acknowledged appropriately?		

Additional comments:

Competence level

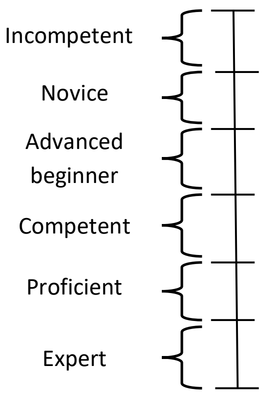

Examples

0	Absence of feature, or highly inappropriate performance
1	Inappropriate performance, with major problems evident
2	Evidence of competence, but lack of consistency
3	Competent, but some inconsistencies
4	Good features, but minor inconsistencies
5	Very good features, very minimal inconsistencies
6	Excellent performance, effective management of difficulties

INTERVENTION FIDELITY TOOL		
PARTICIPANT ID	M S - _ _ - _ _ _	iStep-MS

CONSULTATION THREE: KEEPING UP WITH THE CHANGES	
**Deliverer ID:**	M S D E - _ _ - _ _
**Reviewer (initials):**	
**Date of review:**	D D M M Y Y Y Y

CONTENT OF SESSION	COMPLETED			
	Yes	Partial	No	N/A
1. Review pre-reading and check understanding of what constitutes a set-back for them and the range of possibilities for overcoming a setback.				
2. Opportunity for questions from pre-reading.				
3. Review goals, diary recordings and discuss what went well and what got in the way.				
4. Facilitate discussion on coping with setbacks and identifying strategies.				
5. Introduce pacing; generate pacing tips and plans. Check understanding.				
6. Facilitate identification of own pacing and prioritising tips.				
7. Building habits – generate 'if-then' plans to prevent setbacks or build habits.				
8. Review overall sedentary behaviour and physical activity targets and set new specific goals and record in diary.				
9. Identification of specific barriers and obstacles.				
10. Detail planning – facilitate what, where, when, who, how long? Facilitate problem solving.				
11. Use of confidence ruler and appropriate guidance of goals.				
12. Discuss if participant wishes to set new goals between sessions.				
13. Final check: summarise what has been agreed and check participant understanding.				
14. Plan date/time next session. Remind participant of tasks and to bring workbook.				

INTERVENTION FIDELITY TOOL		
**PARTICIPANT ID**	**M S -** ☐☐ - ☐☐☐	iStep-MS

SKILLS	SCORE (0-6)	COMMENTS
**1. Framing, pacing, focus & use of time**<br>• Did the structure and flow of the session facilitate successful use of time?<br>• Were unproductive digressions steered past sensitively?		
**2. Empowering explanations**<br>• Did explanations invite discussion?		
**3. Collaboration and active listening: therapeutic alliance**<br>• Were open questions used?<br>• Was the participant encouraged to actively lead/ problem solve?<br>• Were goals participant led?		
**4. Goal setting and actions**<br>• Were goals set at 3 levels (PA, step and sedentary)?<br>• Did the action planning facilitate problem solving?		
**5. Feedback, reviewing and summarising**<br>• Was praise used appropriately to support change?<br>• Did feedback reinforce change talk?<br>• Was understanding/meaning checked?		
**6. Building self-efficacy**<br>• Were own examples appropriately encouraged?<br>• Was pre-reading reflection appropriately acknowledged?<br>• Were opportunities to affirm change talk and action taken?		
**Additional comments:**		

Competence level	Examples	
Incompetent	0	Absence of feature, or highly inappropriate performance
Novice	1	Inappropriate performance, with major problems evident
Advanced beginner	2	Evidence of competence, but lack of consistency
Competent	3	Competent, but some inconsistencies
Proficient	4	Good features, but minor inconsistencies
Expert	5	Very good features, very minimal inconsistencies
	6	Excellent performance, effective management of difficulties

INTERVENTION FIDELITY TOOL		
**PARTICIPANT ID**	**M S -** ☐☐ - ☐☐☐	

CONSULTATION FOUR: BUILDING LASTING HABITS	
**Deliverer ID:**	**M S D E -** ☐☐ - ☐☐
**Reviewer (initials):**	
**Date of review:**	D D M M Y Y Y Y

CONTENT OF SESSION	COMPLETED			
	Yes	Partial	No	N/A
1. Review pre-reading and check understanding of tips and techniques, what they have found useful and how they can continue to use these.				
2. Opportunity for questions on pre-reading.				
3. Review goals, diary recordings and discuss what went well and what got in the way.				
4. Review overall progress over the past three months and notice the changes.				
5. Discuss what the Programme meant to the participant personally and encourage self-reflection.				
6. Building habits: discuss methods of maintaining lasting change.				
7. Identify and record the most useful steps / strategies for maintaining / reducing sedentary behaviour and maintaining or increasing physical activity.				
8. Help set target for the next three months and revise use of diary.				

INTERVENTION FIDELITY TOOL	
PARTICIPANT ID	M S - ☐☐ - ☐☐☐

SKILLS	SCORE (0-6)	COMMENTS
**1. Framing, pacing, focus & use of time**<br>• Did the structure and flow of the session facilitate successful use of time?<br>• Were unproductive digressions steered past sensitively?		
**2. Empowering explanations**<br>• Did explanations invite discussion/participation?		
**3. Collaboration and active listening: therapeutic alliance**<br>• Were open questions used?<br>• Was the participant encouraged to actively lead/ problem solve?<br>• Were goals participant led?		
**4. Goal setting and actions**<br>• Was the participant encouraged to review and develop their own future goals independently<br>• Did the action planning facilitate problem solving?		
**5. Feedback, reviewing and summarising**<br>• Was praise used appropriately to support change?<br>• Did feedback reinforce change talk?<br>• Was understanding/meaning checked?		
**6. Building self-efficacy**<br>• Was optional use of forms highlighted?<br>• Were opportunities to affirm change talk and action taken?		

Additional comments:

Competence level	Examples	
Incompetent	0	Absence of feature, or highly inappropriate performance
Novice	1	Inappropriate performance, with major problems evident
Advanced beginner	2	Evidence of competence, but lack of consistency
Competent	3	Competent, but some inconsistencies
Proficient	4	Good features, but minor inconsistencies
Expert	5	Very good features, very minimal inconsistencies
	6	Excellent performance, effective management of difficulties
