# Supplementary material for: Clinical and cost-effectiveness of the iStep-MS physical activity and sedentary behaviour intervention for managing fatigue in people with multiple sclerosis: protocol for a multicentre randomised controlled trial
Source: BMJ Open. 2026 Jul 20;16(7):e121358. doi: 10.1136/bmjopen-2026-121358 (PMC13386072; doi:10.1136/bmjopen-2026-121358)
Supplement: online supplemental file 3 [file bmjopen-16-7-s003.pdf]

FIDELITY TO HANDBOOK CHECKLIST		
PARTICIPANT ID	M S - __ __ - __ __ __	
DATE COMPLETED	D D M M Y Y Y Y	

Assessor completes this information and checklist at the 3 month follow-up.

FIDELITY TO HANDBOOK CHECKLIST	
Assessed by (initials):	

Has the participant's handbook been photographed?	Yes ☐	No ☐

CONSULTATION ONE: FIRST STEPS				
CONTENT OF HANDBOOK	COMPLETED			COMMENTS
	Yes	Partial	No	
Questions and reflections page (p13)	☐	☐	☐	
About you (p14)	☐	☐	☐	
Personal benefits (p16)	☐	☐	☐	
Thoughts on SB and PA (p18)	☐	☐	☐	
Summary of SB and PA from Smartwatch (p20)	☐	☐	☐	
Physical activity journey (p23)	☐	☐	☐	
Diary: sedentary behaviour (p26)	☐	☐	☐	
Diary: PA (p27)	☐	☐	☐	
Diary: step count (p28)	☐	☐	☐	

CONSULTATION TWO: OVERCOMING CHALLENGES				
Content of handbook	COMPLETED			Comments
	Yes	Partial	No	
Questions and reflections page (p35)	☐	☐	☐	
Identifying barriers (p40)	☐	☐	☐	
Ideas for solutions (p40)	☐	☐	☐	
Plan for solutions (p40)	☐	☐	☐	
Diary: sedentary behaviour (p42)	☐	☐	☐	
Diary: PA (p43)	☐	☐	☐	
Diary: step count (p44)	☐	☐	☐	

FIDELITY TO HANDBOOK CHECKLIST		
**PARTICIPANT ID**	**M S -** ☐☐ - ☐☐☐	

CONSULTATION THREE: KEEPING UP WITH THE CHANGES				
**CONTENT OF HANDBOOK**	**COMPLETED**			**COMMENTS**
	**Yes**	**Partial**	**No**	
Questions and reflections page (p51)	☐	☐	☐	
Coping with setbacks (p54, 55)	☐	☐	☐	
Prioritising and pacing tips/plans (p57)	☐	☐	☐	
If-then plans (p58)	☐	☐	☐	
Diary: sedentary behaviour (p60)	☐	☐	☐	
Diary: PA (p61)	☐	☐	☐	
Diary: step count (p62)	☐	☐	☐	

CONSULTATION FOUR: BUILDING LASTING HABITS				
**CONTENT OF HANDBOOK**	**COMPLETED**			**COMMENTS**
	**Yes**	**Partial**	**No**	
Revision exercise (p67)	☐	☐	☐	
Questions and reflections page (p69)	☐	☐	☐	
Reviewing progress (70)	☐	☐	☐	
Meaning of process (p71)	☐	☐	☐	
Advice on habit building (p74)	☐	☐	☐	
New targets: sedentary behaviour (p74)	☐	☐	☐	
New targets: PA (p74)	☐	☐	☐	
New targets: step count (p74)	☐	☐	☐	

ADDITIONAL DOCUMENTS				
	**COMPLETED**			**COMMENTS**
	**Yes**	**Partial**	**No**	
Additional diary sheets (p83-106)	☐	☐	☐	
