# Supplementary material for: Clinical and cost-effectiveness of the iStep-MS physical activity and sedentary behaviour intervention for managing fatigue in people with multiple sclerosis: protocol for a multicentre randomised controlled trial
Source: BMJ Open. 2026 Jul 20;16(7):e121358. doi: 10.1136/bmjopen-2026-121358 (PMC13386072; doi:10.1136/bmjopen-2026-121358)
Supplement: online supplemental file 4 [file bmjopen-16-7-s004.docx]

**iStep-MS Focus group topic guide: treatment group**

Aims:

***In person group and online group: a PowerPoint will be shared with questions listed for each topic.***

Introduction

- Fire exits/alarm, if applicable.
- Purpose of the focus group:
  - to find out your views on the iSTEP-MS programme, how it went for you, whether it made any difference to you, and how it could be improved.
- Important to be as honest as you can so we know what works and what needs to change. You don’t have to answer every question, and can stop whenever you want, for instance if you feel tired – just say.
- Confirm consent to participate in focus group (informed consent form will have been completed prior to focus group) and remind participants that they can withdraw at any time without giving a reason.
- Confirm consent to record the focus group.
- Address the issue of confidentiality – all information collected will be confidential and participant names will not be disclosed in the final report. I hope this encourages you to speak openly and freely.
- The information shared by someone else in this focus group should not be shared with anyone outside of the group.
- Ground rules – please ensure mobile phones are on silent. Please also ensure you avoid interrupting others. Please show respect for others- everyone’s views are of interest and the aim of the discussion is to hear as many different thoughts as possible.
- Ask participants if they have any questions before commencing the focus group.

**1. Participant introductions:**

ICE BREAKER: Researcher and participants introduce themselves to the group one at a time (individuals choose what they say) and if they feel comfortable to share the type of MS symptoms they live with.

**2. Affective Attitude (TFA component 1):**

*“Firstly, we are going to talk about your general thoughts and feeling of the iStep-MS programme.”*

- Did you enjoy engaging with the consultations, deliverer, handbook, Fitbit?
- Were there any parts of the programme that you particularly liked? Why?
- Were there any parts that you particularly disliked? Why?

**3. Burden (TFA component 2):**

*“How much effort was required to participate in iStep-MS?”*

*“Were there any parts of the programme that you feel were easy/not difficult at all? Why?”*

*“Were there any parts that you feel were particularly difficult/burdensome? Why?”*

Prompts

- Attending consultations (*in-person/remote?)
- Tracking goals
- Using the Fitbit
- Using the iStep-MS handbook

**4. Ethicality (TFA component 3):**

*“Did taking part in the iStep-MS programme raise any ethical concerns such as privacy or participant safety?”*

*“Were any specific parts of the programme more or less concerning than others?”*

*“How could these be changed to avoid any concerns in the future?”*

- Attending consultations
- Conversations with the deliverer
- Using the Fitbit
- Using the iStep-MS handbook

**5. Perceived Effectiveness (TFA component 4):**

*“How effective did you think the iStep-MS programme was for a) increasing your physical activity, and b) reducing inactivity/sedentary behaviour.”*

*“Were there any parts that you feel were not so effective? What were they and why?”*

- Attending consultations
- Conversations with the deliverer
- Using the Fitbit
- Using the iStep-MS handbook

**6. Intervention Coherence (TFA component 5):**

*“Was it clear how iStep-MS could help you a) increase your physical activity and b) reduce sedentary behaviour/inactivity?”*

*“If it was clearer, (how) would it have affected your behaviour and engagement with the programme?”*

*“Could we make it clearer to participants?”*

**7. Self-efficacy (TFA component 6):**

*“How confident did you feel that you could do what was required to participate in iStep-MS.”*

*“Were there any parts of the programme that you were not confident about doing? What were they and why?”*

- Attending consultations
- Setting goals
- Using the Fitbit
- Using the iStep-MS handbook

**8. Opportunity Costs (TFA component 7):**

*“Did the* *iStep-MS programme interfere with any of your other priorities or prior commitments”*

“Did anything specific contribute to this interference, e.g. length of programme, amount of content, time taken to attend/do each consultation, tracking and recording goals.”

“What could be changed to minimise this interference?”

**9. General acceptability (TFA component 8)**

*“Overall, how acceptable or unacceptable do you think the iStep-MS programme was to you overall”*

If you had to rate out of 5, 1 being “Unacceptable …” and 5 being ”very acceptable….”

**10. Any additional points:**

- Have you any questions or thoughts to add about the things we have discussed here today?
- Is there anything else you want to say that we have not covered / thought about?

**11****. Close the group / thank you for attending.**

- Readdress the issue of confidentiality – all information collected will be confidential and participant names will not be disclosed in the final report. I hope this encourages you to speak openly and freely.
- The information shared by someone else in this focus group should not be shared with anyone outside of the group.

**iStep-MS Focus group topic guide: intervention deliverers**

Introduction

- Purpose of the focus group:
  - to find out your views on the iStep-MS programme, what went well, what went badly, how it could be improved for the future.
- Important to be as honest as you can so we know what works and what needs to change. You don’t have to answer any question, and can stop whenever you want, for instance if you feel tired – just say.
- We will record the interview so we can listen to you properly. What you say will remain confidential to the research team. Any quotes in reports of the study will be anonymised to protect your identity.
- Please can we have your verbal consent to take part in the focus group.

Experiences in preparation for the iStep-MS study

- Why did you agree to take part in this study?
- Please tell us about any preparation and training you were given to deliver the iStep-MS programme
  - What did it involve?
  - What was useful to you? What wasn’t? and why?
  - What did you think of any training materials or documents you received?
  - How could the preparation or training be improved if we were to do it again?
- What were you hoping for from taking part in the iStep-MS programme?
  - Prompts: goals, hopes for the content and process, how it would be run, personal development
- Did you have any concerns about delivering iStep-MS as in the way that it was planned If so, what were these?

Experience in delivery of iStep-MS

- Tell me about your experiences of delivering the iStep-MS programme
  - What went well?
  - What went less well?
  - Were there any barriers / challenges? How did you address them?
  - What do you think were the key elements of iStep-MS?
  - How well were you able to deliver the key elements of the programme?
- Follow-up if not covered
  - How did the 1:1 sessions go?
  - How well were you able to tailor the programme for individual clients or patients?
    - Agreeing goals and plans
    - Taking account of the impact of MS (e.g. communication, fatigue)
  - What do you think of the programme structure?
    - Workbook content/layout
    - Duration, frequency and number of consultation sessions
    - Length of whole programme
    - What, if anything, do you think needs changing?
  - How was the venue for the consultations? (environment, location, resources, accessibility)
- Did you follow the iStep-MS handbook exactly or adapt it / do different things?
  - If you adapted it, what were the most significant adaptations / changes you made?
- Were the clients or patients suitable for the iStep-MS programme?
  - Prompt: did they have MS-related issues that iStep-MS could help?
  - Would you change the criteria for entry to the programme? If so, how?
- How would you change the iStep-MS programme to make it better?

Acceptability

- How comfortable did you feel delivering iStep-MS? (Affective attitude)
- How much effort did it take you to deliver iStep-MS? (Burden)
  - How much time and resources does it take you to deliver the programme?
  - Would these resources be available for you to continue the programme outside of the project?
- Can you give examples of how the programme either supported or challenged the participants values? (Ethicality)
- How effective did you think iStep-MS was for increasing physical activity and reducing sedentary behaviour? (Perceived effectiveness)
  - What parts were effective?
  - Were there any parts that you feel were not so effective? What were they and why?
  - What improvements could make the invention more deliverable in practice?
- How knowledgeable do you feel now about delivering the programme? (Intervention coherence)
- How confident are you in providing iStep-MS? (Self-efficacy)
- Do you feel that iStep-MS interfered with your other priorities? (Opportunity costs)
- How well aligned was istep-MS to the general aims and processes of your service?
- Overall, how acceptable was the iStep-MS to you? (General acceptability)

Close the group / thank you for attending.
