# Supplementary material for: Clinical and cost-effectiveness of the iStep-MS physical activity and sedentary behaviour intervention for managing fatigue in people with multiple sclerosis: protocol for a multicentre randomised controlled trial
Source: BMJ Open. 2026 Jul 20;16(7):e121358. doi: 10.1136/bmjopen-2026-121358 (PMC13386072; doi:10.1136/bmjopen-2026-121358)
Supplement: Supplementary file 5 [file bmjopen-2026-121358.reviewer_comments.pdf]

PEER REVIEW HISTORY

BMJ Open publishes all reviews undertaken for accepted manuscripts. Reviewers are asked to complete a checklist review form and are provided with free text boxes to elaborate on their assessment. These free text comments are reproduced below.

ARTICLE DETAILS

Title (Provisional)

Clinical and cost-effectiveness of the iStep-MS physical activity and sedentary behaviour intervention for managing fatigue in people with multiple sclerosis: Protocol for a multi-centre randomised controlled trial

Authors

Bailey, Daniel; Norris, Emma; LeWarne, Myles D; Cerexhe, Luke; Anokye, Nana; Banstola, Amrit; Gwatsvaira, Joe; Norris, Meriel; Ryan, Jennifer; Stuart, Beth; Thomson, Ann; Kilbride, Cherry

---

This article was not externally reviewed at BMJ Open. Protocols that have been independently assessed prior to submission to BMJ Open are usually fast-tracked to publication on the grounds that further substantial changes will not be possible. This independent assessment will usually be external, independent review for both a substantial grant award from a non-commercial or government funder and ethics approval.
